# Supplementary material for: Serum vitamin E levels and chronic inflammatory skin diseases: A systematic review and meta-analysis
Source: PLoS One. 2021 Dec 14;16(12):e0261259. doi: 10.1371/journal.pone.0261259 (PMC8670689; doi:10.1371/journal.pone.0261259)
Supplement: S1 Table — (DOCX) [file pone.0261259.s002.docx]

**S1 Table. Systematic search strategy**

| Database | Strategy |
| --- | --- |
| Pubmed | (“Dermatitis, Atopic”[Mesh]OR atopic dermat*[tiab] OR “vitiligo”[Mesh] OR vitiligo*[tiab] OR “psoriasis”[Mesh]OR psoriasis[tiab] OR “acne”[Mesh] OR acne*[tiab]) AND (“Vitamin E”[Mesh] OR Vitamin E[tiab] OR “tocopherol”[Mesh] OR tocopherol[tiab]) |
| Scopus | ((TITLE-ABS-KEY(“atopic dermat*”)) OR ((TITLE-ABS-KEY(“vitiligo”)) OR ((TITLE-ABS-KEY(“psoriasis”)) OR ((TITLE-ABS-KEY(“acne”))) AND ((TITLE-ABS-KEY(“Vitamin E”)) OR ((TITLE-ABS-KEY(“tocopherol”))) |
| Web of Science | (TS=“atopic dermat*” OR TS=“vitiligo” OR TS=“psoriasis” OR TS=“acne”) AND (TS=“Vitamin E” OR TS=“tocopherol”) |
