# Supplementary material for: Serum vitamin E levels and chronic inflammatory skin diseases: A systematic review and meta-analysis
Source: PLoS One. 2021 Dec 14;16(12):e0261259. doi: 10.1371/journal.pone.0261259 (PMC8670689; doi:10.1371/journal.pone.0261259)
Supplement: S2 Table — (DOCX) [file pone.0261259.s003.docx]

| **Skin diseases** | **Study** | **Group** | | **Age** | **Sex (M:F)** | **Duration of**  **the disease** | **Serum vitamin E levels** | **Unit of vitamin E** |
| --- | --- | --- | --- | --- | --- | --- | --- | --- |
| Vitiligo | Ines et al.,2006 | Control | | 31.5(21-45) | 15:25 | NA | 9.18±1.56 | μg/mL |
|  |  | Patients | Stable | 31.7 (18-59) | 6:12 | 11.5 | 9.61±1.39 |  |
|  |  |  | Active | 32.5(19-66) | 10:8 | 8.5 | 9.24±1.29 |  |
|  | Khan et al., 2009 | Control | | NA | NA | NA | 1.45±0.26 | mg/dL |
|  |  | Patients | | NA | NA | NA | 0.46±0.15 |  |
|  | Agrawal et al., 2014 | Control | | NA | NA | NA | 0.66±0.15 | mg/dL |
|  |  | Patients | | NA | NA | NA | 0.67±0.22 |  |
|  | Jain et al., 2008 | Control | | NA | 1:1 | - | 1.13±0.57 | mg/100 ml |
|  |  | Patients | | NA | 1:1 | NA | 0.7±0.43 |  |
|  | Dell'Anna et al., 2001 | Control | | NA | NA | NA | 0.36±0.12 | ng /mg protein |
|  |  | Patients | Stable | 42( 18-53) | 27:13 | NA | 0.21±0.15 |  |
|  |  |  | Active |  |  | NA | 0.18±0.16 |  |
|  | Agrawal et al., 2004 | Control | | 5-15 | NA | NA | 9.22±1.84 | μg/ml |
|  |  | Patients | |  |  |  | 8.13±1.44 |  |
|  |  | Control | | 16-25 | NA | NA | 8.09±2.09 |  |
|  |  | Patients | |  |  |  | 8.49±2.39 |  |
|  |  | Control | | 26-35 | NA | NA | 9.2±1.25 |  |
|  |  | Patients | |  |  |  | 8.25±1.25 |  |
|  |  | Control | | 36-45 | NA | NA | 8.96±1.94 |  |
|  |  | Patients | |  |  |  | 8.86±2.94 |  |
|  | Picardo et al., 1994 | Control | | NA | NA | NA | 11.1±1.18 | μg/ml |
|  |  | Patients | | 19-45 | 37:25 | NA | 10.61±2.49 |  |
| Psoriasis | Kökçam et al., 1999 | Control | | NA | NA | NA | 9.3±3.81 | μg/ml |
|  |  | Patients | | 9-76 | 20:14 | NA | 8.32±3.75 |  |
|  | Pereira et al., 2004 | Control | | 47.4 ± 13.3 | 55:45 | NA | 13.2±3.1 | nmol g^-1^ Hb |
|  |  | Patients | Mild | 45.9 ± 12.2 | 57:43 | NA | 12.3±2.3 |  |
|  |  |  | Severe |  |  | NA | 11.4±2.4 |  |
|  |  |  | Mild+ Severe |  |  | NA | 11.9±2.5 |  |
|  | Jain, et al., 1988 | Control | | NA | NA | NA | 8.1±0.65 | mg/L |
|  |  | Patients | Grade I | NA | NA | NA | 8.6±0.41 |  |
|  |  |  | Grade II | NA | NA | NA | 4.8±0.21 |  |
|  |  |  | Grade III | NA | NA | NA | 3.1±0.31 |  |
|  |  |  | Grade I+II+III | NA | NA | NA | 6.3±0.60 |  |
|  | Demir et al., 2013 | Control | | 39.23 ± 15.32 | 10:21 | NA | 27.19±8.89 | μmol/L |
|  |  | Patients | | 38.46 ± 10.32 | 13:24 | 7.74 ± 7.68 | 26.21±5.13 |  |
|  | Pujari et al., 2014 | Control | | NA | NA | NA | 10.64±0.22 | mg/Lit |
|  |  | Patients | Mild | 20- 60 | NA | NA | 9.87 ± 0.16 |  |
|  |  |  | Severe |  | NA | NA | 8.28 ± 0.12 |  |
|  | Severin et al., 1999 | Control | | NA | NA | NA | 27.17±5.09 | μmol/l |
|  |  | Patients | | NA | NA | NA | 35.54±6.72 |  |
| Atopic dermatitis | Oh et al., 2010 | Control | | NA | NA | NA | 16.52±12.09 | μmol/L |
|  |  | Patients | | NA | NA | NA | 13.66±8.95 |  |
|  | [Sivaranjani](https://www.ncbi.nlm.nih.gov/pubmed/?term=Sivaranjani%20N%5BAuthor%5D&cauthor=true&cauthor_uid=24551611) et al., 2013 | Control | | NA | NA | NA | 1.46±0.14 | mg% |
|  |  | Patients | | 35 (10-60) | NA | NA | 0.94±0.24 |  |
|  | Daniluk et al., 2019 | Control | | 5.5 (2–15) | 14:8 | NA | 10.69±3.64 | μmol/l |
|  |  | Patients | Mild | 6 (1–15) | 8:12 | NA | 7.9±1.77 |  |
|  |  |  | Severe | 9 (3–13) | 4:5 | NA | 9.65±2.13 |  |
|  |  |  | Mild+Severe | 6 (1–15) | 12:17 | NA | 8.83±2.1 |  |
|  | Hozyasz et al., 2004 | Control | | 1-9 | NA | NA | 3.17±0.49 | Mmol/L |
|  |  | Patients | | 1-9 | NA | NA | 2.14±0.69 |  |
| Acne | El-akawi et al., 2006 | Control | | 21.3 ± 5.3 | NA | NA | 5.4 ± 2.1 | mg/L |
|  |  | Patients | | 21.0 ± 5.4 | NA | NA | 5.9 ± 1.4 |  |
|  | Ozuguz et al., 2013 | Control | | 30.45±9.46 | 12:44 | NA | 11.06±3.08 | mg/L |
|  |  | Patients | Mild | 28.54±8.30 | 33:61 | NA | 8.28±3.12 |  |
|  |  |  | Severe |  |  | NA | 5.66±2.93 |  |
|  |  |  | Mild+Severe |  |  | NA | 7.88±3.00 |  |
|  | Tunçez Akyürek et al.,2020 | Control | | 19.7 ± 2.49 | NA | NA | 11.81 ± 3.46 | mg/L |
|  |  | Patients | Mild | 18.67 ± 3.36 | NA | NA | 10.9 ± 2.46 |  |
|  |  |  | Severe |  | NA | NA | 10.64 ± 1.99 |  |
|  |  |  | **Moderate** |  | NA | NA | 10.97 ± 2.68 |  |
|  |  |  | Mild+**Moderate+**Severe |  | NA | NA | 10.84 ± 2.37 |  |
