# Supplementary material for: Serum vitamin E levels and chronic inflammatory skin diseases: A systematic review and meta-analysis
Source: PLoS One. 2021 Dec 14;16(12):e0261259. doi: 10.1371/journal.pone.0261259 (PMC8670689; doi:10.1371/journal.pone.0261259)
Supplement: S3 Table — (DOCX) [file pone.0261259.s004.docx]

| Study | Selection | | | | Comparability | | Outcome | | | Total stars |
| --- | --- | --- | --- | --- | --- | --- | --- | --- | --- | --- |
|  | Is the case definition adequate? | Representativeness of the cases | Selection of Controls | Definition of Controls | Comparability of cohorts  on the basis of the design or analysis(for most important factor) | Comparability of cohorts  on the basis of the  design or analysis(for any additional factor) | Ascertainment of exposure | Same method of ascertainment for cases and controls | Non-Response rate |  |
| Oh et al., 2010 | ***** |  | ***** | ***** | ***** | ***** | ***** | ***** | ***** | **8** |
| [Sivaranjani](https://www.ncbi.nlm.nih.gov/pubmed/?term=Sivaranjani%20N%5BAuthor%5D&cauthor=true&cauthor_uid=24551611) et al., 2013 | ***** |  | ***** | ***** | ***** |  | ***** | ***** |  | **6** |
| Daniluk et al., 2019 | ***** | ***** | ***** | ***** | ***** | ***** | ***** | ***** |  | **8** |
| Hozyasz et al., 2004 | ***** |  | ***** | ***** | ***** |  | ***** | ***** |  | **6** |
| Ines et al.,2006 |  |  | ***** | ***** | ***** |  | ***** | ***** |  | **5** |
| Khan et al., 2009 | ***** |  |  | ***** | ***** |  | ***** | ***** |  | **5** |
| Agrawal et al., 2014 | ***** | ***** | ***** | ***** | ***** | ***** | ***** | ***** |  | **8** |
| Jain et al., 2008 | ***** |  |  | ***** | ***** |  | ***** | ***** |  | **5** |
| Dell'Anna et al., 2001 | ***** |  |  | ***** | ***** |  | ***** | ***** |  | **5** |
| Agrawal et al., 2004 | ***** |  |  | ***** | ***** | ***** | ***** | ***** |  | **6** |
| Picardo et al., 1994 | ***** |  |  | ***** | ***** |  | ***** | ***** |  | **5** |
| Kökçam et al., 1999 | ***** |  |  | ***** | ***** |  | ***** | ***** |  | **5** |
| Pereira et al., 2004 | ***** |  | ***** | ***** | ***** | ***** | ***** | ***** |  | **7** |
| Jain, et al., 1988 |  | ***** |  | ***** | ***** |  | ***** | ***** |  | **5** |
| Demir et al., 2013 | ***** | ***** |  | ***** | ***** | ***** | ***** | ***** |  | **7** |
| Pujari et al., 2014 | ***** |  |  | ***** | ***** | ***** | ***** | ***** |  | **6** |
| Severin et al., 1999 | ***** |  | ***** | ***** | ***** |  | ***** | ***** |  | **6** |
| Ozuguz et al., 2013 | ***** |  | ***** | ***** | ***** | ***** | ***** | ***** |  | **7** |
| Z. El-akawi  Et al.,2006 | ***** |  | ***** | ***** | ***** | ***** | ***** | ***** |  | **7** |
| Tuncez Akyurek et al., 2020 | ***** | ***** | ***** | ***** | ***** | ***** | ***** | ***** |  | **8** |
