# Supplementary material for: Serum vitamin E levels and chronic inflammatory skin diseases: A systematic review and meta-analysis
Source: PLoS One. 2021 Dec 14;16(12):e0261259. doi: 10.1371/journal.pone.0261259 (PMC8670689; doi:10.1371/journal.pone.0261259)
Supplement: S4 Table — (DOCX) [file pone.0261259.s005.docx]

| Skin diseases | Trial characteristic | Number of studies | SMD (95% CI) | *P* | |
| --- | --- | --- | --- | --- | --- |
|  |  |  |  | *P* _h_^1^ | *P* _h_^2^ |
| Vitiligo | Study quality  ≥6 stars  <6 stars | 2  5 | -0.14(-0.47, 0.20)  -1.06(-1.86, -0.26) | =0.188  <0.001 | 0.250 |
|  | Region  Asia  Europe | 5  2 | -0.65(-1.32, 0.02)  -0.88(-1.63, -0.14) | <0.001  =0.001 | 0.792 |
|  | Matched  Yes  No | 7  0 | -0.70 (1.21, -0.19)  NA | <0.001  NA | NA |
|  | Age  Children  Other | 2  5 | -0.38(-1.27, 0.50)  -0.74(-1.43, -0.14) | =0.001  <0.001 | 0.685 |
|  | Gender  Female > Male  Female = Male | 5  2 | -0.57(-1.18, 0.05)  -1.07(-1.38, -0.76) | <0.001  0.419 | 0.555 |
|  | Sample size  ≥100  <100 | 3  4 | -0.15(-0.40, 0.10)  -1.22(-2.22, -0.22) | 0.231  0.001 | 0.161 |
| Psoriasis | Study quality  ≥6 stars  <6 stars | 4  2 | -2.09(-3.70, -0.48)  -2.90(-5.03, -0.77) | <0.001  <0.001 | 0.710 |
|  | Region  Asia  Europe | 4  2 | -3.78(-5.78, -1.79)  -0.02(-0.92, 0.89) | <0.001  <0.001 | 0.084 |
|  | Matched  Yes  No | 5  1 | -2.04(-3.32, -0.76)  1.41(0.88, 1.94) | <0.001  NA | 0.361 |
|  | Age  Children  Other | 0  6 | NA  -2.37( -3.57 to -1.18) | NA  <0.001 | NA |
|  | Gender  Female > Male  Female < Male  Female = Male  No data | 1  2  1  2 | -0.13(-0.61, 0.35)  -0.43(-0.67, -0.18)  -3.78(-7.12, -0.43)  -4.65(-10.25, 0.96) | NA  0.712  NA  <0.001 | 0.237 |
|  | Sample size  ≥100  <100 | 4  2 | -1.66(-3.02, -0.30)  -3.24(-5.39, -1.10) | <0.001  <0.001 | 0.575 |
| Atopic dermatitis | Study quality  ≥6 stars  <6 stars | 4  0 | -0.89(-1.52 to -0.26)  NA | <0.001  NA | NA |
|  | Region  Asia  Europe | 2  2 | -1.42(-3.75, 0.92)  -0.71(-1.42, -0.00) | <0.001  <0.001 | 0.435 |
|  | Matched  Yes  No | 4  0 | -0.89(-1.52, -0.26)  NA | <0.001  NA | NA |
|  | Age  Children  Other | 3  1 | -0.62(-1.13, -0.10)  -2.65(-3.41, -1.88) | <0.001  NA | NA |
|  | Gender  Female > Male  Female < Male  No data | 1  1  2 | -0.68(-1.05, -0.30)  -0.26(-0.47, -0.06)  -2.19(-3.01, -1.36) | NA  NA  0.086 | NA |
|  | Sample size  ≥100  <100 | 1  3 | -0.26(-0.47, -0.05)  -1.02(-1.83, -0.21) | NA  <0.001 | NA |
| Acne | Study quality  ≥6 stars  <6 stars | 3  0 | -0.67( -1.05, -0.30)  NA | NA  NA | NA |
|  | Region  Asia  Europe | 3  0 | -0.67( -1.05, -0.30)  NA | NA  NA | NA |
|  | Matched  Yes  No | 3  0 | -0.67( -1.05, -0.30)  NA | NA  NA | NA |
|  | Age  Children  Other | 0  3 | NA  -0.67( -1.05, -0.30) | NA  NA | NA |
|  | Gender  Female > Male  No data | 1  2 | -1.26(-1.77, -0.72)  -0.31(-0.50, -0.13) | NA  0.991 | NA |
|  | Sample size  ≥100  <100 | 3  0 | -0.67( -1.05, -0.30)  NA | NA  NA | NA |

*^1^P* _h_ values were for heterogeneity within a subgroup.

*^2^P* _h_ values were for heterogeneity between subgroups by meta-regression.
