# Supplementary material for: Serum vitamin E levels and chronic inflammatory skin diseases: A systematic review and meta-analysis
Source: PLoS One. 2021 Dec 14;16(12):e0261259. doi: 10.1371/journal.pone.0261259 (PMC8670689; doi:10.1371/journal.pone.0261259)
Supplement: Attachment — Submitted filename: Response to Reviewers.docx [file pone.0261259.s006.docx]

**The responses to the academic editor’ comments (Journal Requirements) are as following:**

**Comment 1:** Please ensure that your manuscript meets PLOS ONE's style requirements, including those for file naming. The PLOS ONE style templates can be found at

https://journals.plos.org/plosone/s/file?id=wjVg/PLOSOne_formatting_sample_main_body.pdf and

<https://journals.plos.org/plosone/s/file?id=ba62/PLOSOne_formatting_sample_title_authors_affiliations.pdf>

**Response:** Thank you for your helpful suggestion. We have modified our format according to PLOS ONE's style requirements.

**Comment 2:** Please consider modifying your title to ensure that it is specific, descriptive, concise, and comprehensible to readers outside the field (for example by clarifying the research question that this study aims to answer).

**Response:** Thank you for your helpful suggestion. We had modified our title as “Serum vitamin E levels and chronic inflammatory skin diseases: A systematic review and meta-analysis”. We hope it is comprehensible to readers now.

**Comment 3:** We note that the grant information you provided in the ‘Funding Information’ and ‘Financial Disclosure’ sections do not match.

When you resubmit, please ensure that you provide the correct grant numbers for the awards you received for your study in the ‘Funding Information’ section.

**Response:** Thank you for your helpful suggestion. We had corrected the grant Information.

**Comment** 4: Thank you for stating the following in the Acknowledgments Section of your manuscript:

“The study was funded by Higher education reform project of 2018 (Liaoning, China)”

We note that you have provided additional information within the Acknowledgements Section that is not currently declared in your Funding Statement. Please note that funding information should not appear in the Acknowledgments section or other areas of your manuscript. We will only publish funding information present in the Funding Statement section of the online submission form.

Please remove any funding-related text from the manuscript and let us know how you would like to update your Funding Statement. Currently, your Funding Statement reads as follows:

“The funders had no role in study design, data collection and analysis, decision to publish, or preparation of the manuscript.”

Please include your amended statements within your cover letter; we will change the online submission form on your behalf.

**Response:** Thank you for your helpful suggestion. We had removed funding-related text from the manuscript. We are sorry for our incorrect information. We corrected it as following: This work was supported by the Basic Research Projects of Colleges and Universities of Liaoning Province, China (LQ2017037).

**Comment** 5: In your Data Availability statement, you have not specified where the minimal data set underlying the results described in your manuscript can be found. PLOS defines a study's minimal data set as the underlying data used to reach the conclusions drawn in the manuscript and any additional data required to replicate the reported study findings in their entirety. All PLOS journals require that the minimal data set be made fully available. For more information about our data policy, please see http://journals.plos.org/plosone/s/data-availability.

Upon re-submitting your revised manuscript, please upload your study’s minimal underlying data set as either Supporting Information files or to a stable, public repository and include the relevant URLs, DOIs, or accession numbers within your revised cover letter. For a list of acceptable repositories, please see http://journals.plos.org/plosone/s/data-availability#loc-recommended-repositories. Any potentially identifying patient information must be fully anonymized.

Important: If there are ethical or legal restrictions to sharing your data publicly, please explain these restrictions in detail. Please see our guidelines for more information on what we consider unacceptable restrictions to publicly sharing data: http://journals.plos.org/plosone/s/data-availability#loc-unacceptable-data-access-restrictions. Note that it is not acceptable for the authors to be the sole named individuals responsible for ensuring data access.

We will update your Data Availability statement to reflect the information you provide in your cover letter.

**Response:** Thank you for your helpful suggestion. All relevant data and the minimal data set are available in Supporting Information files, and our Data Availability statement has been provided in our cover letter.

**Comment 6:** Please ensure that you refer to Figure 2, 3, 4 and 5 in your text as, if accepted, production will need this reference to link the reader to the figure.

**Response:** Thank you for your helpful suggestion. References of Figure 2, 3, 4 and 5 should be in our text, and were added in the corresponding positions in the revised version.

**Comment 7:** We note you have included a table to which you do not refer in the text of your manuscript. Please ensure that you refer to Table 5 in your text; if accepted, production will need this reference to link the reader to the Table.

**Response:** Thank you for your helpful suggestion. We are sorry for our Table reference missing. References of Figures and Tables were corrected and added in the corresponding positions in the revised version.

**Comment 8:** Please include your tables as part of your main manuscript and remove the individual files. Please note that supplementary tables (should remain/ be uploaded) as separate "supporting information" files.

**Response:** Thank you for your helpful suggestion. Tables have been added in our main manuscript; supplementary tables have been uploaded as separate "supporting information" files in our revised version.

**The responses to the Reviewer are as following:**

**Comments to the Author**
1. Is the manuscript technically sound, and do the data support the conclusions?
The manuscript must describe a technically sound piece of scientific research with data that supports the conclusions. Experiments must have been conducted rigorously, with appropriate controls, replication, and sample sizes. The conclusions must be drawn appropriately based on the data presented.

Reviewer #1: No

Reviewer #2: Partly

Reviewer #3: Yes

2. Has the statistical analysis been performed appropriately and rigorously?

Reviewer #1: Yes

Reviewer #2: Yes

Reviewer #3: Yes

3. Have the authors made all data underlying the findings in their manuscript fully available?
The [PLOS Data policy](http://www.plosone.org/static/policies.action#sharing) requires authors to make all data underlying the findings described in their manuscript fully available without restriction, with rare exception (please refer to the Data Availability Statement in the manuscript PDF file). The data should be provided as part of the manuscript or its supporting information, or deposited to a public repository. For example, in addition to summary statistics, the data points behind means, medians and variance measures should be available. If there are restrictions on publicly sharing data—e.g. participant privacy or use of data from a third party—those must be specified.

Reviewer #1: Yes

Reviewer #2: Yes

Reviewer #3: Yes

4. Is the manuscript presented in an intelligible fashion and written in standard English?
PLOS ONE does not copyedit accepted manuscripts, so the language in submitted articles must be clear, correct, and unambiguous. Any typographical or grammatical errors should be corrected at revision, so please note any specific errors here.

Reviewer #1: No

Reviewer #2: Yes

Reviewer #3: No

5. Review Comments to the Author

Please use the space provided to explain your answers to the questions above. You may also include additional comments for the author, including concerns about dual publication, research ethics, or publication ethics. (Please upload your review as an attachment if it exceeds 20,000 characters)

**Reviewer #1: General remark
Comment 1:** English remains a concern, and several minor shortcomings regarding grammar and punctuation must be polished with a revised version. Remember that a flawless manuscript is the task of all co-authors, having read and approved the submission.

**Response:** Thank you very much for reviewing our manuscript and giving us so many useful suggestions. Your comments have helped us to improve our article significantly. We highlighted all changes in yellow in our revised manuscript.

We have carefully checked and corrected the grammar and punctuation errors with a revised version, and we hope that the quality of the article has been improved. For improving language, the paper had been revised by an editor of American Journal Experts (AJE).

**Comment 2** **(Abstract):**
**Comment 2.1:** Please note the word maximum which is allowed with PLOS ONE (see Guidelines for Authors), to increase your information regarding your results, and to allow future readers to switch to your full text.

**Response:** Thank you for your helpful suggestion. Our revised abstract is 199 words, which is fewer than the previous one.

**Comment 2.2:** Your aim was "To clarify the serum level of vitamin E in chronic inflammatory dermatosis, (...)." And you have concluded that "low serum Vitamin E levels increased the susceptibility risk of certain chronic inflammatory skin diseases." This would not seem convincing. Indeed, this would even seem meaningless. With your Conclusions, please stick exclusively to your aims. Do not simply repeat your results here. Do not provide banalities (known from each and every other paper). Instead, provide a reasonable and generalizable extension of your outcome.

**Response:** Thank you for your helpful suggestion. We rewrote the Conclusions in "Abstract" as following: "Our meta-analysis showed that serum vitamin E levels were lower in patients suffering from vitiligo, psoriasis, atopic dermatitis and acne. This study highlights the need to evaluate vitamin E status to improve its level in patients with skin diseases." This change is marked yellow in the revised manuscript.

**Comment 3 (Intro)**

**Comment 3.1:** "Skin disease is one of the most common human illnesses." How can you say this? Please have a close look on caries, and on periodontitis. Again, please avoid common phrases seen with almost every paper.

**Response:** Thank you for your helpful suggestion. We are sorry for our common phrases in paper. We have corrected it in our revised manuscript.

**Comment 3.2:** Please revise for referencing according to Journal style. "(...) pruritus, and so on[1, 2]." must read "(...) pruritus, and so on[1, 2]." First, make use of your spacebar, and revise thoroughly. Second, what do you mean when referring to "and so on"?

**Response:** Thank you for your helpful suggestion. We are sorry for us inaccurate use of words. We corrected this sentence in our revised manuscript. The spacebars have been revised thoroughly.

**Comment 3.3:** Do not repeat statements. "(...) the relationship between serum vitamin E level and skin diseases is still unclear." and "However, the existing studies produced conflicting results." would provide comparable (or even the same) information. Please revise carefully, to facilitate reading.

**Response:** Thank you for your helpful suggestion. We have rewritten this part of introduction, we hope its better.

**Comment 3.4:** "So far, researches on level of serum vitamin E and skin diseases mainly focus on chronic inflammation skin diseases like vitiligo, psoriasis, atopic dermatitis and acne." References missing.

**Response:** Thank you for your nice suggestion. The references of the sentence have been added in our revised manuscript.

**Comment 3.5:** Please clarify whether there already been any "systematic reviews of literature and meta-analyses" referring to vitamin E and dermal diseases. Make clear what you will add to the literature.

**Response:** Thank you for your helpful suggestion. There is lack of meta-analysis on the relationship between serum levels of vitamin E and chronic inflammatory skin diseases. We have added this information in our revised manuscript.

**Comment 3.6**: Moreover, you surely had some idea prior to starting your study. What was your null hypothesis. Remember that H0 must be deducible from the foregoing thoughts. Revise carefully.

**Response:** Thank you for your helpful suggestion. Our H0 could be “the increased risk of chronic inflammatory skin diseases in some populations may partly be explained by their poor serum vitamin E status”.

**Comment 4 (Meths):
Comment 4.1**: Heading must read "Materials and methods". Again, you are strongly encouraged to consult the Journal's guidelines. Additionally, double checking some recently published PLOS ONE papers will help.

**Response:** Thank you for your helpful suggestion. According to your suggestion, we have carefully checked and modified our papers consult the Journal’s guidelines.

**Comment 4.2**: "(...) by two authors (Qi Lan and Mengxin Luo)." must read "(...) by two authors (Q.L. and M.L.). Same with "C.Z.".

**Response:** Thank you for your helpful suggestion. According to your suggestion, we have corrected the abbreviation form of authors’ name in revised version.

**Comment 4.3**: "The literature search strategy was showed in Table. 1." must read "The literature search strategy is given with Table 1.". Revise carefully, to avoid any typos/delete full stops not considered necessary.

**Response:** Thank you for your helpful suggestion. According to your suggestion, we have corrected the “Table. 1.” to “Table 1.” in revised version.

**Comment 4.4**: "(version 13, StataCorp LP, College Station, TX)." must read "(Stata Statistical Software, Release 13; StataCorp LP, College Station, TX, USA)."

**Response:** Thank you for your helpful suggestion. According to your suggestion, we have corrected the “(version 13, StataCorp LP, College Station, TX).” to “(Stata Statistical Software, Release 13; StataCorp LP, College Station, TX, USA).” in revised version.

**Comment 5 (Results):**

**Comment 5.1:** "In quality assessment, scores of all included case-control studies are showed in Table 3" Please clarify the quality grades with your text. Remember that only high-quality papers should be included, to avoid repetition of any "conflicting results" (which have been deemed by the Authors). Please clarify, and discuss.

**Response:** Thank you for your helpful suggestion. The Newcastle–Ottawa Scale (NOS) was used to assess the quality of non-randomized studies. Using this scale, every individual study is judged on eight items, categorized into three dimensions: selection of study; comparability; and ascertainment of exposure/outcome. Stars are given for each quality item and the highest quality studies are given up to nine stars. A study is considered of high-quality if there are 3 or 4 stars in selection domain AND 1 or 2 stars in comparability domain AND 2 or 3 stars in exposure/ outcome domain^1,2^. Studies with ≥ 6 stars, 4–6 stars were considered high and moderate quality, respectively. Studies with ≤ 4 stars were considered low quality and were excluded^3,4^. According to you suggestion, we have clarified the quality grades with our revised manuscript.

1. Wells GA, Shea B, O’Connell D et al. The Newcastle–Ottowa Scale (NOS) for assessing the quality of nonrandomised studies in meta-analysis: Ottawa Hospital Research Institute; 2014URL http://www.ohri.ca/programs/clinical_epidemiology/oxford.asp (last accessed June 2018)
2. Benatar JR, Stewart RAH. [Cardiometabolic risk factors in vegans; A meta-analysis of observational studies.](https://pubmed.ncbi.nlm.nih.gov/30571724/) PLoS One. 2018 Dec 20;13(12):e0209086.
3. Founou RC, Founou LL, Essack SY. Clinical and economic impact of antibiotic resistance in developing countries: A systematic review and meta-analysis..PLoS One. 2017 Dec 21;12(12):e0189621.
4. Liu T, Zhong S, Liao X, Chen J, He T, Lai S, Jia Y. [A Meta-Analysis of Oxidative Stress Markers in Depression.](https://pubmed.ncbi.nlm.nih.gov/26445247/) PLoS One. 2015 Oct 7;10(10):e0138904.

We more objectively described the quality of the included studies: As assessed by the Newcastle-Ottawa Scale (NOS), thirteen studies were of high quality, and the remaining seven studies were medium quality. For selection of study groups, 11 of the 20 studies scored 3 or 4 stars. For comparability, all of the 20 studies scored 1 or 2 stars. For assessment of outcome, all of the 20 studies provided ascertainment of exposure, and had same method of ascertainment for cases and controls, but only one study provided non-response rates or described non-respondents. These details have been added in our revised manuscript.

**Comment 5.2:** "(P < 0.00)" would seem unclear. As a general rule, please always provide p values on a 3-digit basis. Format must be "(p = 0.838)", or "(p < 0.001)". Note that lower case "p" would fit to the Journal style.

**Response:** Thank you for your helpful suggestion. We have corrected the format of p values in the revised manuscript.

**Comment 5.3:** High heterogeneity would seem a problem, and there must be a convincing rationale to proceed with your paper. Please discuss.

**Response:** Thank you for your helpful suggestion. Between-study heterogeneity is common in meta-analysis and it is essential to explore the potential sources of between-study heterogeneity. Therefore, we conducted subgroup and meta-regression analyses on potential confounders including study quality, region, study size, age, and whether sex or age-matched of studies to explore the potential sources of between-study heterogeneity. However, these factors were not found to be sources of heterogeneity in our meta-analysis, but other possibilities related to skin diseases, such as variations in lifestyle and dietary practices, cannot be ruled out for contribution to heterogeneity.

Furthermore, sensitivity analysis was performed to try to assess the influence of individual studies on the overall effect size. Our findings did not change significantly with the removal of any of the studies. Therefore, sensitivity analyses revealed that the results produced in this meta-analysis were robust.

**Comment 5.4:** "As a sensitivity analysis, a meta-influence plot was used to analyze the influence of individual studies on the overall effect size (Figure. 7)." This would not seem acceptable. Please remember that it is considered the task of the Authors to guide the reader. Simply referring to a Table, or to a Figure (without providing any explanations) is not deemed professional.

**Response:** Thank you for your helpful suggestion. In a sensitivity analysis, a meta-influence plot was used to analyze the influence of individual studies on the overall effect size. As shown in Fig 7, no single study has influence on the above-mentioned pooled effect of the association between serum vitamin E and skin diseases, further confirmed the robust of our findings. The description of sensitivity analysis has been added in revised version.

**Comment 6 (Disc):**

**Comment 6.1:** Refer to H0 with the first paragraph of this section.

**Response:** Thank you for your helpful suggestion. H0 has been added in the first paragraph of this section.

**Comment 6.2:** "It was found that >60% of adults have vitamin E intakes below the EAR (<12 mg/d) in the United States." Again, each and every statement calls for reference(s). Revise thoroughly throughout your text.

**Response:** Thank you for your helpful suggestion. The references of the sentence have been added in our revised manuscript. According to your suggestion, we have checked and revised thoroughly throughout our text.

**Comment 6.3:** "In our study, the lower serum vitamin E levels in patients of vitiligo, psoriasis, atopic dermatitis and acne compared with controls." Please double check and revise sentence.

**Response:** Thank you for your helpful suggestion. We have revised this sentence to “In our study, there were lower serum vitamin E levels in patients with vitiligo, psoriasis, atopic dermatitis and acne than in controls.”

**Comment 6**.**4**: This section has not been thoroughly elaborated, and there would seem room for more profound discursive aspects.

**Response:** Thank you for your helpful suggestion. We have rewritten the discussion, and we hope it’s better.

**Comment 7 (Concl)**

**Comment 7.1:** Please see comments given above. Again, do not simply repeat your results here - these already have been presented (and should be thoroughly discussed, see comments given above). Instead, provide a reasonable and generalizable extension of your outcome.

**Response:** Thank you for your helpful suggestion. We have rewritten the conclusion in our revised version. We hope it’s better.

**Comment 7.2:** Same with "It might be due to the number of researches on (...)." Explanations (and even speculations) are welcome, but must be provided with the Disc section.

**Response:** Thank you for your helpful suggestion. Explanations have been changed to the Disc section.

**Comment 7.3:** Same with "The low quality and high heterogeneity of some included studies means that our results must be interpreted with caution (...)." Again, this is not considered a Conclusion. You should discuss these aspects at the right place. Why did you include those poor papers? Why did you finish such a study involving several poor papers? Please provide a sound and reasonable rationale. Moreover (and again), this is a clear limitation, and the Authors must thoroughly clarify, why PLOS ONE should proceed with this draft. At the end of the day, this only will be another paper considered "unclear", "controversial", or "conflicting". Please see comments given above.

**Response:** Thank you for your helpful suggestion. We are really sorry that we confused the discussion with the conclusion before. The high heterogeneity of our study has been explained in discussion of the revised version.

For the quality of our included study, we shouldn't describe it subjectively before. The quality scale has been described in Materials &Methods, and the quality score of the studies has been presented in result with our revised version. All the changes were marked yellow.

**Comment 7.4:** "More studies of high-quality observational are required to confirm the association between low serum vitamin E levels and skin diseases." Again, this is a meaningless platitude. And, moreover, this would clarify that your study did not reveal any associations, right?

**Response:** Thank you for your helpful suggestion. We are sorry for our not good description. We rewrote these sentences to “Our review is an important finding in terms of future randomized controlled trials which might reveal a direct cause and effect relationship between vitamin E intake as well as serum vitamin E and the risk of skin diseases. Furthermore, potential mechanism associated with preventive effects of vitamin E should be more elucidated in further studies.” We hope it’s better.

**Comment 8 (Refs):**

**Comment 8:** Please revise for uniform formatting. Consult some previously published PLOS ONE papers.

**Response:** Thank you for your helpful suggestion. We have revised reference formatting Consult previously published PLOS ONE papers.

**Reviewer #2:** This systematic review focuses on the relation between vitamin E levels and chronic inflammatory dermatoses.

Thank you very much for reviewing our manuscript. Your comments have helped us to improve our article. We highlighted all changes in yellow.

**Comment 1:** It is widely recognised that it would be better to study dietary patterns rather than single nutrients since nutrients and foods may interact in their biological effects.

**Response:** Thank you for your helpful suggestion. This could be one limitation in our study; therefore we added some descriptions in our Discussion as follows: The reported associations may be confounded by other nutrients due to the synergistic or antagonistic effects of nutrients, and/or lifestyle factors known to influence the risk of skin diseases. Future large-scale studies are warranted to explore such combined effects.

Furthermore, the importance of dietary pattern was highlighted (before referring vitamin E) in our discussion as follows:

Apart from immune disorders/autoimmunity, oxidative stress and genetic predisposition, environmental risk factors including lifestyle behaviors are the main determinants of skin diseases, especially dietary patterns. An increasing body of research indicates that dietary changes may play an important role in chronic skin diseases, especially nutrients that have bioactive functions such as antioxidant or anti-inflammatory activities. It has also been found that dietary habits play an important role in pathogenesis and treatment of chronic skin diseases including psoriasis, vitiligo, acne and atopic dermatitis.

All modifications were marked yellow.

**Comment 2：**Reverse causation is a major issue and should be clearly discussed in the study. Reverse causation can occur when people change their diet or other lifestyle habit after developing a disease or perhaps after having a close family member suffer an event like vitiligo or other immune-related conditions.

**Response:** Thank you for your helpful suggestion. According to your suggestion, we discussed on the reverse causation concluded from our meta-analysis as the following two points: 1) the importance of maintaining serum vitamin E levels might be suggested for patients diagnosed with skin diseases. 2) Improving vitamin E levels in patients with skin diseases might have a positive effect. Please see the details:

Many studies of dietary interventions on skin diseases have proven that nutrient supplementation is an auxiliary means for the treatment of skin diseases. [Elgoweini](https://pubmed.ncbi.nlm.nih.gov/?term=Elgoweini+M&cauthor_id=19553407) et al. founded that vitamin E supplementation prevented lipid peroxidation in the cellular membrane of melanocytes and increased the effectiveness of NB-UVB in patients with stable vitiligo. [Goforoushan](https://pubmed.ncbi.nlm.nih.gov/?term=Goforoushan+F&cauthor_id=24498826) et al. reported that vitamin E prevents dermal complications of isotretinoin. Oh et al. observed a lower likelihood of atopic dermatitis in children who consumed more dietary vitamin E, which led to higher serum vitamin E levels. Therefore, the importance of maintaining serum vitamin E levels might be suggested for patients diagnosed with skin diseases.

Some clinical studies have been investigated as a mainstay treatment of vitamin E in skin diseases. Two randomized, double-blind, placebo-controlled clinical trials (RCTs) using the same model performed by Javanbakht et al. and Jaffary et al. concluded that alpha tocopherol increased erythrocyte superoxide dismutase activity and reducted the Scoring Atopic Dermatitis index in patients with atopic dermatitis. A clinical trial conducted by Tsoureli-Nikita et al. showed that in patients with atopic dermatitis, treatment with oral vitamin E (400 IU/day) for 8 months had observable reductions in skin lesions and pruritus, comparable to treatment with topical corticosteroid and oral antihistamines. One RCT performed by [Kharaeva](https://pubmed.ncbi.nlm.nih.gov/?term=Kharaeva+Z&cauthor_id=19041224) et al. found that supplementation with antioxidants containing vitamin E resulted in the significant improvement of clinical conditions and normalization of oxidative stress markers in patients with severe erythrodermic and arthropathic forms of psoriasis. Improving vitamin E levels in patients with skin diseases might have a positive effect. However, much more research is needed on this subject due to the small amount of currently published studies.

All modifications were marked yellow.

**Reviewer #3**: This is a commendable review by the authors to understand how vitamin E can relate to various skin issues. There are a couple revisions that are needed before this article can be considered for acceptance:

Thank you for your kind comments. They helped us to improve the quality of our manuscript.

**Comment 1：** The English is poor. I can't detail every single grammatical error as they are numerous. Please have a native English speaker review this for grammatical accuracy.

**Response:** Thank you for your helpful suggestion. We are sorry for our poor English. For improving language, the paper had been revised by an editor of American Journal Experts (AJE). We have checked and corrected the grammatical errors in a revised version, and we hope that the quality of the article has been improved.

**Comment 2：** The authors should not refer to vitamin E as if it is just one molecule. Please detail the subsets of vitamin E to detail what was actually measured. Was it alpha tocopherol, gamma tocopherol, tocotrienol subvariants, etc?

**Response:** Thank you for your helpful suggestion. It is necessary to add the details of vitamin E subsets. In our study, thirteen studies elaborated that alpha tocopherol was estimated in their investigations. However, the other seven studies did not describe the subunit of measured vitamin E. We have added this information both in “Result” and “Table 1”. The changes were marked yellow.
