# Supplementary material for: Serum vitamin E levels and chronic inflammatory skin diseases: A systematic review and meta-analysis
Source: PLoS One. 2021 Dec 14;16(12):e0261259. doi: 10.1371/journal.pone.0261259 (PMC8670689; doi:10.1371/journal.pone.0261259)
Supplement: Attachment — Submitted filename: Response to Reviewers.docx [file pone.0261259.s007.docx]

**The response to the Journal Requirements is as following:**

Please review your reference list to ensure that it is complete and correct. If you have cited papers that have been retracted, please include the rationale for doing so in the manuscript text, or remove these references and replace them with relevant current references. Any changes to the reference list should be mentioned in the rebuttal letter that accompanies your revised manuscript. If you need to cite a retracted article, indicate the article’s retracted status in the References list and also include a citation and full reference for the retraction notice.

**Response:** Thank you very much for your suggestion. We examined the references carefully and found one retracted article (Sato et al., 2007). We replaced it by a study of Kwan et al. in 2018. This change was marked yellow in “References”.

**The responses to the Reviewer are as following:**

Reviewers' comments:

Reviewer's Responses to Questions

**Comments to the Author**

1. If the authors have adequately addressed your comments raised in a previous round of review and you feel that this manuscript is now acceptable for publication, you may indicate that here to bypass the “Comments to the Author” section, enter your conflict of interest statement in the “Confidential to Editor” section, and submit your "Accept" recommendation.

Reviewer #1: All comments have been addressed

Reviewer #3: All comments have been addressed

Reviewer #4: All comments have been addressed

2. Is the manuscript technically sound, and do the data support the conclusions?

The manuscript must describe a technically sound piece of scientific research with data that supports the conclusions. Experiments must have been conducted rigorously, with appropriate controls, replication, and sample sizes. The conclusions must be drawn appropriately based on the data presented.

Reviewer #1: Yes

Reviewer #3: Yes

Reviewer #4: Yes

3. Has the statistical analysis been performed appropriately and rigorously?

Reviewer #1: Yes

Reviewer #3: Yes

Reviewer #4: Yes

4. Have the authors made all data underlying the findings in their manuscript fully available?

The [PLOS Data policy](http://www.plosone.org/static/policies.action#sharing) requires authors to make all data underlying the findings described in their manuscript fully available without restriction, with rare exception (please refer to the Data Availability Statement in the manuscript PDF file). The data should be provided as part of the manuscript or its supporting information, or deposited to a public repository. For example, in addition to summary statistics, the data points behind means, medians and variance measures should be available. If there are restrictions on publicly sharing data—e.g. participant privacy or use of data from a third party—those must be specified.

Reviewer #1: Yes

Reviewer #3: Yes

Reviewer #4: Yes

5. Is the manuscript presented in an intelligible fashion and written in standard English?

PLOS ONE does not copyedit accepted manuscripts, so the language in submitted articles must be clear, correct, and unambiguous. Any typographical or grammatical errors should be corrected at revision, so please note any specific errors here.

Reviewer #1: Yes

Reviewer #3: Yes

Reviewer #4: Yes

6. Review Comments to the Author

Please use the space provided to explain your answers to the questions above. You may also include additional comments for the author, including concerns about dual publication, research ethics, or publication ethics. (Please upload your review as an attachment if it exceeds 20,000 characters)

Reviewer #1: With the help of the reviewers, this revised and re-sublmitted draft has been considerably improved, and would seem ready to be forwarded to the external referees.

**Response:** Thank you for reviewing my manuscript and helping me improve the quality of my article.

Reviewer #3: Thank you for the edits and this is suitable for publication. The authors now delineate the different subsets of vitamin E more clearly and the English is improved.

**Response:** Thank you very much for your affirmation of our work.

Reviewer #4: In this study, a comprehensive systematic review and meta analysis was conducted to evaluate the effects of vitamin e levels on chronic inflammatory skin diseases in human subjects. This review is well written. Also, in the first revise, the reviewer's comments were responded appropriately. To the reviewer's knowledge, there has been no systematic review on this topic in the past, and it will provide important knowledge and useful information for this research area. However, there are some comments that could elevate the quality of this paper. Reviewer added recommendations and suggestions regarding this.

**Response:** Thank you very much for reviewing our manuscript and your kind comments. They helped us to improve our manuscript. We highlighted all changes in yellow.

**Comment 1:** This review focuses primarily on chronic inflammatory skin diseases. Are there any reported synergies between chronic inflammatory skin diseases and other physiological effects (such as lifestyle disease and immunologic disease)? It would be more interesting for the reader to add these explanations in the first half of the article.

**Response:** Thank you for your helpful suggestion. There are many studies reported that chronic inflammatory skin diseases were associated and often coexisting with obesity, cardiovascular disease diabetes mellitus and many other immune-related clinical conditions. We have added these explanations in the first paragraph of “Introduction”.

**Comment 2:** Reviewer concern about the subjects’ characteristics used for this analysis. Authors mentioned about their age but less information about their sex even though some investigations the number of male/ female is considerably biased. Was the sex a considerable factor?

**Response:** Thank you for your helpful suggestion. It is necessary to add the details on sex. In our study, 13 of the 20 studies presented the information on sex. However, the other seven studies did not describe. We have added information about sex in “Table 1”. The changes were marked yellow.

Among these studies with descriptions on sex, some studies have more men than women and some have more women than men. Therefore, sex was a considerable factor for our analysis. Subgroup and meta-regression analyses were performed on sex to explore whether sex was the potential source of between-study heterogeneity. The result showed that sex was not found to be a source of heterogeneity in our meta-analysis. We have added these details in “Results”, “Discussion” and “S4 Table” The changes were marked yellow (In manuscript “gender” was used).

**Comment 3:** Reviewer felt the resolution of each figure was low. Reviewer recommends that the author replace it with a higher resolution figure.

**Response:** Thank you for your helpful suggestion. We have replaced all figures with higher resolution ones.

**Comment 4:** The authors state that the effects of vitamin E on chronic inflammatory skin diseases are predominantly antioxidant and anti-inflammatory. On the other hand, for example, in the following review reported this year, Reflections on a century of vitamin E research: Looking at the past with an eye on the future, Free Radical Biology and Medicine, Volume 175, 2021, Pages 155-160, ISSN [0891-5849](tel:0891-5849), doi.org/10.1016/j. freeradbiomed.2021.07.042 .https://www.sciencedirect.com/science/article/pii/S0891584921007048?via%3Dihub

These review mention that vitamin E's antioxidant and anti-inflammatory effects do not work in the body. What do the authors think of such a debate?

**Response:** Thank you for your helpful suggestion. We have carefully read the article you recommended, it is time to renew our opinion that as follows: 1. the antioxidant and anti-inflammatory effects of vitamin E on diseases (including chronic inflammatory skin diseases) are most studied in the past, but many evidences has been described that the oxidative event and anti-inflammatory action were not primarily correlated with vitamin E.

2. When it comes to the effect of vitamin E, it does not refer to all subtypes of vitamin E. Such as α-tocopherol structure is unique and not substitutable with other tocopherols or tocotrienols. For example, in cell cultures and animal models the effects of α-tocopherol were not duplicated by β-tocopherol, α- and γ-tocopherol (provided with essentially equal antioxidant properties) modulate very differently gene expression in murine T lymphocyte.

3. There are many possible mechanisms like gene expression regulation, microRNA modifications, tocopherol associated proteins, albumin binding and stem cell differentiation, as well as active forms of vitamin E including tocopheryl phosphate and vitamin E metabolites could be involved in vitamin E’s benefit, which we should pay attention now as well as explore in future.

Therefore, we have attenuated the statement emphasizing antioxidant and anti-inflammatory effects of vitamin E in our “Introduction”.

We also looked up studies on the possible mechanisms of vitamin E and skin diseases, however, there is a lack of information on mechanisms like gene expression regelation, microRNA modifications, tocopherol associated proteins, albumin binding and stem cell differentiation. At present, inflammation and oxidative damage play important roles in chronic inflammatory skin diseases, so we still focus on these two points in first part of “Discussion”. Furthermore, we emphasize the importance of vitamin E subtypes and their role in skin diseases, and future research is needed to achieve a better understanding of the possible molecular events leading to gene regulation, microRNA modifications, tocopherol associated proteins, albumin binding and stem cell differentiation by vitamin E in skin diseases, as well as its active form in body. We marked these changes in yellow in our revised manuscript.
